# Electronic Structure and Optical Properties of Tin Iodide Solution Complexes

**DOI:** 10.1021/acs.jpca.3c01754

**Published:** 2023-05-12

**Authors:** Freerk Schütt, Ana M. Valencia, Caterina Cocchi

**Affiliations:** † Institute of Physics, Carl-von-Ossietzky Universität Oldenburg, 26129 Oldenburg, Germany; ‡ Center for Nanoscale Dynamics, Carl-von-Ossietzky Universität Oldenburg, 26129 Oldenburg, Germany; ¶ Physics Department and IRIS Adlershof, Humboldt-Universität zu Berlin, 12489 Berlin, Germany

## Abstract

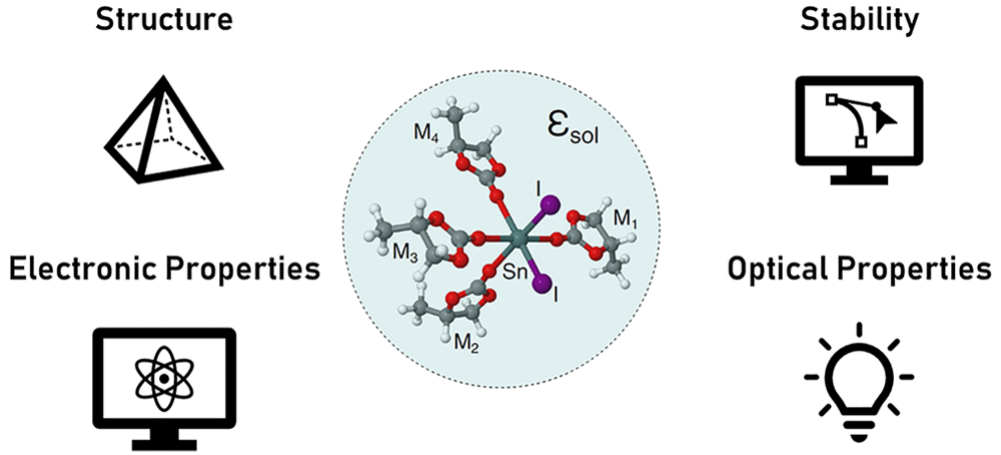

The emerging interest in tin halide perovskites demands a robust understanding of the fundamental properties of these materials starting from the earliest steps of their synthesis. In a first-principles work based on time dependent density functional theory, we investigate the structural, energetic, electronic, and optical properties of 14 tin iodide solution complexes formed by the SnI_2_ unit tetracoordinated with molecules of common solvents, which we classify according to their Gutmann’s donor number. We find that all considered complexes are energetically stable and their formation energy expectedly increases with the donating ability of the solvent. The energies of the frontier states are affected by the choice of solvent, with their absolute values decreasing with the donor number. The occupied orbitals are predominantly localized on the tin iodide unit, while the unoccupied ones are distributed also on the solvent molecules. Owing to this partial wave function overlap, the first optical excitation is generally weak, although the spectral weight is red-shifted by the solvent molecules being coordinated to SnI_2_ in comparison to the reference obtained for this molecule alone. Comparisons with results obtained on the same level of theory on Pb-based counterparts corroborate our analysis. The outcomes of this study provide quantum-mechanical insight into the fundamental properties of tin iodide solution complexes. This knowledge is valuable in the research on lead-free halide perovskites and their precursors.

## Introduction

Tin halide perovskites are emerging materials for photovoltaic and optoelectronic applications,^1−3^ responding to the pressing quest for nonpolluting and nontoxic compounds for solar cells.^4,5^ Like their Pb-based counterparts, these systems are produced via solution chemistry, and the efficiency of the resulting thin films crucially depends on the physicochemical properties of the precursors.^6−8^ The choice of the solvent is a particularly critical point as it can induce oxidation^9−11^ and thus contribute to the rapid degradation of the material in operational conditions.^6,12,13^ As a result of these efforts, it is now common knowledge that the popular solvent dimethyl sulfoxide (DMSO) is detrimental for the stability of tin halide perovskite solar cells.^6,8^ This finding has stimulated the search for guiding principles toward the choice of optimal solvents for the synthesis of this class of materials.^14,15^

The missing piece of the puzzle is a systematic, first-principles analysis of the microscopic properties of tin halide solution complexes as building blocks for corresponding perovskite structures. Similar studies performed on lead halide counterparts have significantly contributed to disclosing the characteristics of these compounds^16−21^ and, hence, to better understanding the evolution process of these systems from solution precursors to thin films through several intermediate steps.^22,23^ In the case of Sn-based halide perovskites, such efforts have been conducted primarily in conjunction with experiments.^6^ A dedicated ab initio study is, to the best of our knowledge, still missing.

In the framework of time-dependent density functional theory coupled to the polarizable continuum model (PCM), we investigate the physical properties of 14 solution complexes, SnI_2_M_4_, where M indicates a common solvent molecule chosen among compounds previously adopted experimentally in the synthesis of tin halide precursors.^6^ In the presented analysis, we focus on the structural, energetic, electronic, and optical properties of these systems. We characterize the considered compounds by looking at bond lengths and angles and by comparing them with their counterparts in PbI_2_M_4_ complexes. We assess their formation energy and rationalize our findings with respect to the donor number of the solvent. Finally, we inspect the electronic structure in order to interpret their absorption spectra and disclose the characteristics of their optical excitations, including their composition and spatial distribution among the constituents.

## Computational Methods

**Figure 1:**
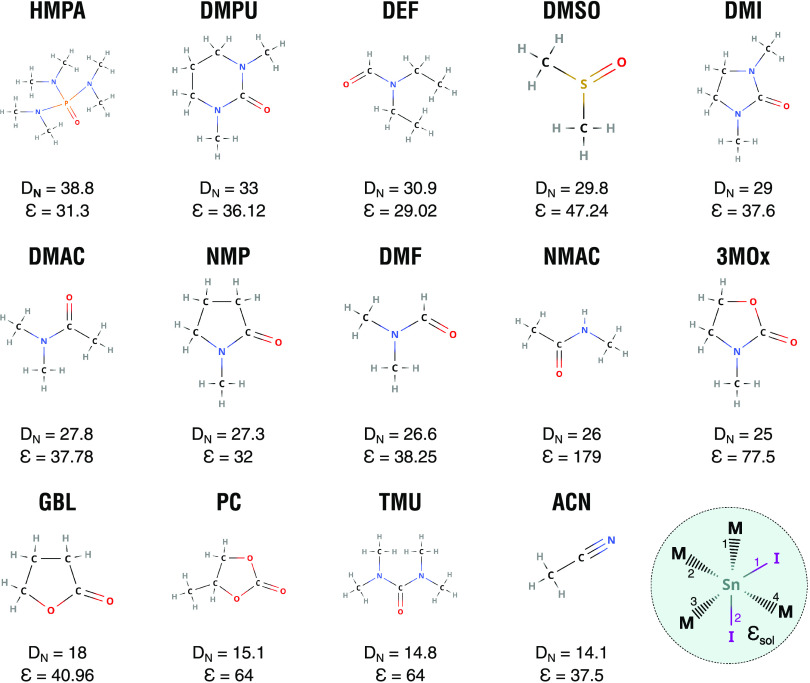
Overview of the 14 solvents considered in this study with their respective Gutmann’s donor number (*D*_*N*_) and static dielectric constant (ε): hexamethylphosphoramide (HMPA), *N*,*N*′-dimethylpropyleneurea (DMPU), *N*,*N*-diethylformamide (DEF), dimethyl sulfoxide (DMSO), 1,3-dimethyl-2-imidazolidinone (DMI), dimethylacetamide (DMAC), *N*-methyl-2-pyrrolidone (NMP), dimethylformamide (DMF), *N*-methylacetamide (NMAC), 3-methyl-2-oxazolidinone (3MOx), γ-butyrolactone (GBL), propylene carbonate (PC), tetramethylurea (TMU), and acetonitrile (ACN). On the bottom right panel, a sketch of the SnI_2_M_4_ compounds in the solvent cavity is reported: the numbers label the Sn–I (purple) and the Sn–M (black) bonds.

All ab initio calculations presented in this study were performed using density functional theory (DFT)^24,25^ as implemented in the Gaussian 16 software package.^26^ The implicit solvent interactions were simulated with the PCM^27,28^ adopting the values for the dielectric constants of the solvents listed in Figure 1. van der Waals interactions were accounted for using the semiempirical Grimme D3 dispersion scheme.^29^ Reference calculations on the isolated SnI_2_ complex assumed to be implicitly solvated in DMSO were performed adopting the PCM.

The geometries of the SnI_2_M_4_ complexes were optimized through the minimization of the interatomic forces. By means of additional frequency calculations, we checked that all the obtained structures represented global minima except for SnI_2_(TMU)_4_ and SnI_2_(HMPA)_4_, which exhibited one and four negative frequencies, respectively. We argue that this result does not impact the following analysis of the electronic and optical properties of the complexes, which is primarily focused on identifying trends. For these optimizations, we used the generalized gradient approximation in the Perdew–Burke–Ernzerhof (PBE) parametrization^30^ together with the LANL2DZ basis set; pseudopotentials were adopted for Sn and I atoms and the double-ζ basis set cc-pVDZ for the remaining lighter species. We checked that the choice of this functional, which is substantially more efficient than hybrid approximations and adopted in previous studies on similar systems,^16−18,20,21^ did not affect the electronic structure of the resulting optimized structures. For single-point-geometry DFT calculations, for the population and natural bond order (NBO) analysis, as well as for the time-dependent DFT (TDDFT) runs, we adopted the range-separated hybrid functional CAM-B3LYP^31^ in combination with the SDD basis set together with the corresponding SDD pseudopotentials for Sn and I and the quadruple-ζ cc-pVQZ basis set for the lighter atoms. The output of (TD)DFT calculations was postprocessed to obtain relevant information on the systems. The molecular orbitals (MOs) of the SnI_2_M_4_ complexes were analyzed with the natural atomic orbital (NAO) method as implemented in the Multiwfn^32^ software on the basis of the NBO calculations performed with Gaussian 16.^33,34^

The properties of the complexes were analyzed in light of the Gutmann donor number (*D*_*N*_) of the solvents as the primary classifier according to their Lewis basicity. This property is recognized as a determining factor for the effectiveness of the solvents and specifically for their ability to solvate halide perovskite precursors.^22,35−37^ The values of *D*_*N*_ adopted in this work were taken from available references and, in the absence of the latter, estimated from first-principles from the ionization potential (IP) and the electron affinity (EA) of the compounds as computed from DFT^38,39^ (see Figure 1 and Table S1 in the Supporting Information). The absorption spectra evaluated from TDDFT+PCM are plotted including a Lorentzian broadening of 70 meV for the excitation line widths.

## Systems

In this study, we consider 14 tin iodide solution complexes characterized by a SnI_2_ backbone in the axial configuration surrounded by four solvent molecules binding to it (see Figure 1). The initial geometries are set up based on earlier works on lead iodide solution complexes,^6,17,20^ while the choice of solvents is based on experimental work on tin halide perovskite precursors.^6^ We consider the common solvents propylene carbonate (PC), which is a green compound, and DMSO as a point of reference for comparisons with Pb-based counterparts. In fact, DMSO leads to the oxidation of Sn^2+^ and, as such, is detrimental to tin halide film formation.^10,40^ Other chosen solvents belong to the class of amide (DEF, DMF, DMAC, NMAC, NMP) and diamide (TMU, DMI, and DMPU). The remaining ones are representative of carbamate (3MOx), of solvents containing the P=O bond (HMPA), and of species dissolving from formaldiminium iodide (GBL). Finally, we consider acetonitrile (ACN), which binds to SnI_2_ via a nitrogen atom, in contrast to all the other solvents mentioned above which bind to a metal halide unit via an oxygen (see Figure 1). The SnI_2_M_4_ compounds are simulated atomistically in order to assess the quantum-mechanical interactions holding them together. The additional electrostatic forces coming from the surrounding solvent medium are accounted for implicitly within the PCM cavity (see Figure 1, bottom right panel).

## Results and Discussion

### Structural Properties

**Figure 2:**
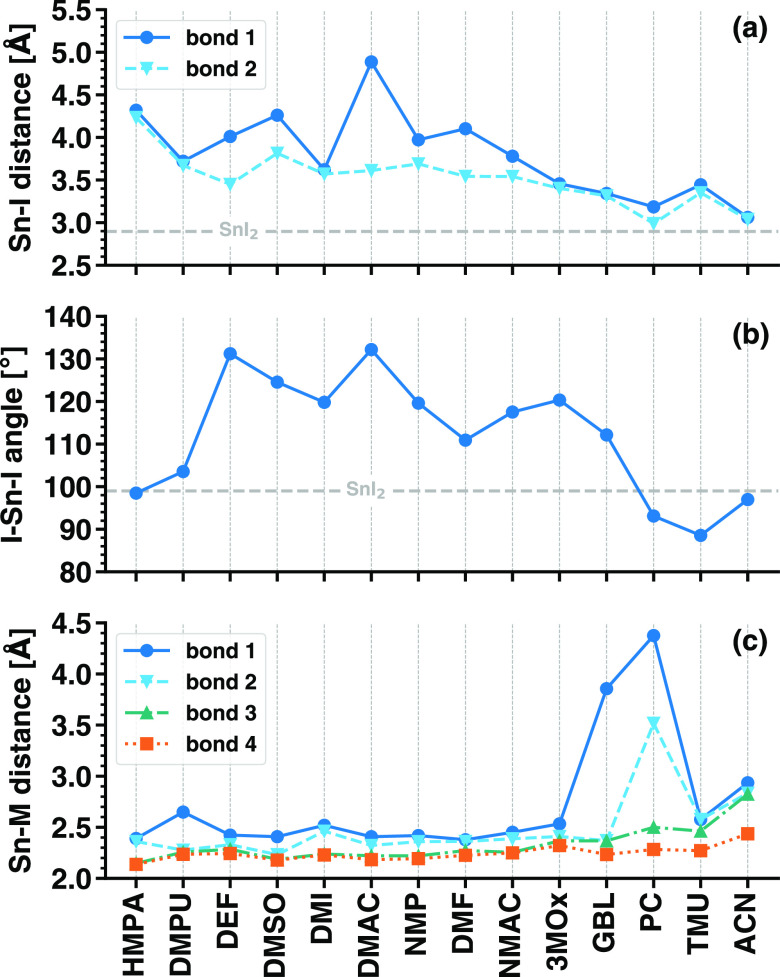
Structural properties of the SnI_2_M_4_ complexes, including (a) the Sn–I distances, (b) the I–Sn–I angle, and (c) the distances between Sn and the anchoring atom of the solvent molecule M (Sn–M distance). The solvents are ordered on the *x*-axis with respect to their donor number *D*_*N*_, decreasing from left to right. The horizontal dashed lines in panels (a) and (b) mark the reference value obtained for SnI_2_.

We start our analysis from the structural properties of the 14 SnI_2_M_4_ complexes considered in this work. Specifically, we inspect the distances between Sn and I atoms in the metal halide backbone, the I–Sn–I angle, and the separation between the Sn atoms and the solvent molecules. Regardless of the solvent, we notice at a glance that the Sn–I bond length is systematically increased in the solution complexes with respect to the SnI_2_ molecule; see Figure 2a. The bond elongation is enhanced by solvents with a large donor number; in fact, in the presence of weakly donating solvents such as PC or ACN, the Sn–I distances remain very close to the reference values obtained for SnI_2_. In some cases, the interactions with solvent molecules induce asymmetric distortions in the tin iodide backbone. In particular, the result obtained with DMAC sticks out. In this case, the SnI_2_ unit is significantly stretched, with one I atom at approximately 3.6 Å apart from Sn and the other one at almost 5 Å. In this configuration, the Sn and I atoms interact only weakly with arguably only one chemical bond remaining in place. As we will see in the following, this characteristic has remarkable consequences on the electronic and optical properties of the complex.

Inspection of the trends for the I–Sn–I angle provides additional indications on the structural changes induced by the solvent molecules on SnI_2_ (see Figure 2b). Despite the elongation of the Sn–I bond length, interaction with HMPA does not lead to any variation of the I–Sn–I angle. The presence of DMPU, ACN, and PC does not strongly modify this structural parameter either. On the other hand, the increase in the Sn–I separation is accompanied by a sizable increase in the I–Sn–I angle in the presence of solvents with intermediate values of *D*_*N*_. The angle tends to close up with weakly donating solvents such as PC, TMU, and ACN, although variations above 10° with respect to the reference value in SnI_2_ can be appreciated only with TMU.

Finally, we analyze the separations between tin atoms in SnI_2_ and the surrounding solvent molecules. As shown in Figure 2c, in most cases, the four Sn–M distances within a complex are almost equal, ranging from 2.1 to 2.6 Å. Notice that the average of these values, namely, 2.3 Å, is very close to the tabulated experimental value for the Sn–O bond length (2.2 Å)^41^ as well as to DFT results obtained for the same quantity in tin oxide.^42^ Yet, a couple of outliers can be identified: Sn–O distances above 3.5 Å testify that SnI_2_ does not bind with one GBL molecule and with two PC ones. In SnI_2_(PC)_4_, we ascribe it to the weak ability of the solvent to bind to tin iodide, where, indeed, the Sn–I separation remains almost intact with respect to the isolated SnI_2_ unit (Figure 2a). Finally, the larger Sn–M distances found in SnI_2_(ACN)_4_ are likely the result of the reduced binding ability of ACN in comparison with solvents with higher *D*_*N*_ that are anchored to SnI_2_ via an oxygen atom. In fact, reference DFT values for Sn–N distances are between 2.2 and 2.3 Å,^43^ namely, very similar to Sn–O separations.

Overall, the structural trends displayed in Figure 2 can be rationalized with the coordinating ability of the solvents. Compounds with high *D*_*N*_ values strongly coordinate with the metal halide salts, competing against I^–^ for the coordination sites around the central metal cation. This is reflected in the larger distances between the metallic cation (Sn^2+^) and the I^–^ anions and smaller distances between Sn^2+^ and the solvent molecules. On the other hand, solvents with lower *D*_*N*_ have a weaker coordination ability with the cation, leading to smaller Sn–I distances and larger Sn–M separations.^22,37^

**Figure 3:**
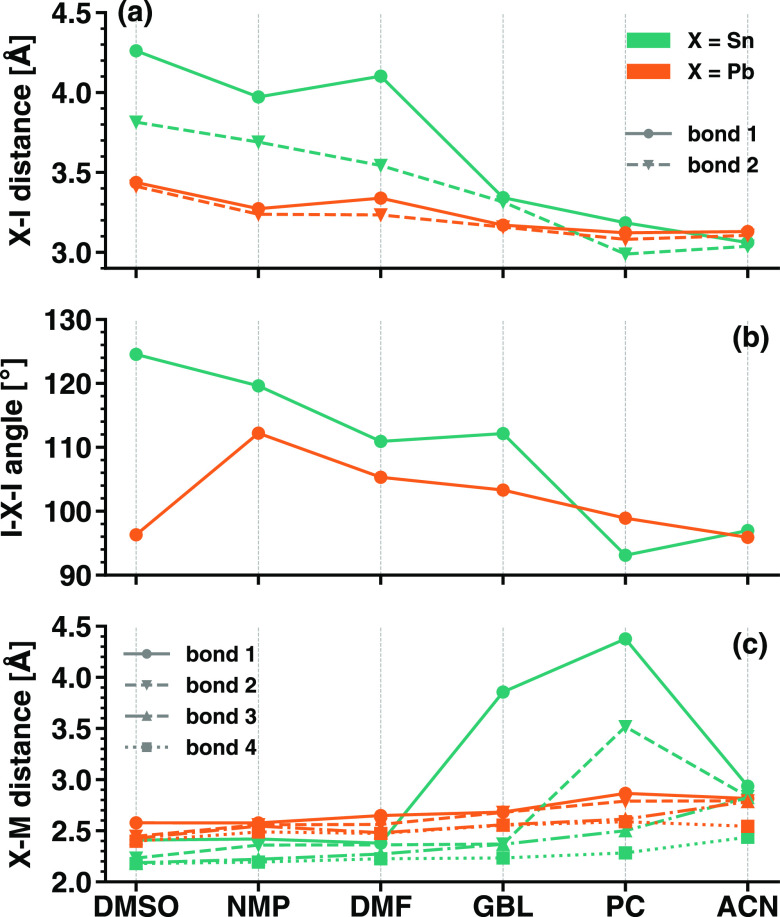
Structural properties of six SnI_2_M_4_ complexes compared to their PbI_2_M_4_ counterparts calculated with identical computational parameters in ref (20): (a) metal–iodine (X–I) distances, (b) metal–iodide (I–X–I) angle, and (c) metal–solvent (X–M) distances are shown. Solvents with decreasing donor number are displayed on the *x*-axis from left to right.

The structural properties of six SnI_2_M_4_ complexes considered in this work can be directly compared to their PbI_2_M_4_ counterparts previously investigated on the same level of theory.^20^ The above-discussed trends of increasing metal–I distance and decreasing I–metal–I angle and metal–M distances for decreasing *D*_*N*_ are common to both sets of complexes (see Figure 3). In the presence of solvents with high *D*_*N*_, such as DMSO, NMP, and to some extent DMF, Sn–I distances are larger than their Pb–I counterparts by more than 0.5 Å. Notice that the distance from I is almost identical for Sn and Pb in the SnI_2_ and PbI_2_ molecules, respectively; see Figure 2a and ref (20). With decreasing solvent donor numbers, Sn–I and Pb–I distances approach each other until they become almost identical with the solvents PC and ACN. Overall we notice more symmetric structures in the Pb-based complexes, as testified by the very similar Pb–I bond lengths regardless of the solvents (see Figure 3a). In contrast, Sn coordinates differently with the two I atoms likely due to its smaller size compared to both Pb and iodine itself. This characteristic can be associated with the tendency to lattice distortion exhibited by bulk tin-based halide perovskites.^44^

Moving on to the angles of the metal–iodide backbone, we notice that those in the tin-based complexes are systematically larger than those in the Pb-based compounds; the only exception is for the compounds formed with PC (Figure 3b). We correlate this trend with the one discussed above for the metal–iodine distances: large bond lengths lead to an opening of the angle and vice versa. Lastly, the Pb–M distances are consistently larger than the Sn–M distances by approximately 0.2 Å and are also more homogeneous among each other (Figure 3c). In contrast to SnI_2_(GBL)_4_ and SnI_2_(PC)_4_, where one and two molecules, respectively, do not bind to the metal–iodide center, in the Pb-based counterparts all solvents are coordinated with PbI_2_.^20^ In general, the choice of the solvent seems to have a larger influence on the structure of the Sn-based complexes compared to the Pb-based ones. The stronger solvent–metal coordination and subsequent weakening of the metal–I interaction appear more significant in Sn-based complexes compared to the Pb-based ones. The structural variability of tin iodide complexes can be ascribed to the small size of Sn compared to Pb, which likely has a more stabilizing influence as the central cation and shows generally stronger coordination.

We conclude this analysis by commenting that the SnI_2_M_4_ complex that is structurally most similar to its PbI_2_M_4_ counterpart is the one including ACN (Figure 3). We interpret this behavior based on the weakest ability of this solvent to coordinate with the metal–iodide center in comparison with the other ones considered in this work. In contrast, the largest structural differences are seen between SnI_2_(DMSO)_4_ and PbI_2_(DMSO)_4_. This result is in line with recent findings relating structural distortions occurring in Sn halide perovskites synthesized with DMSO due to solvent-induced oxidation of tin.^6,8,10,40^

### Energetic Stability

With the insight gained from the analysis of the structural properties, we move on to the investigation of the energetic stability of the SnI_2_M_4_ complexes. The formation energy, *E*_f_, is calculated as
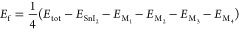
1where *E*_tot_ is the total energy of the complex in its optimized structure,  is the energy of the SnI_2_ molecule in the solution complex, and  is the energy of each of the four solvent molecules in their relaxed geometry within the complex.^20^ In eq 1, the energy contributions from all four solvent molecules are considered separately, taking into account the differences induced in their geometries when they bind to SnI_2_. Higher (lower) stabilities are indicated by more (less) negative values of *E*_f_.

**Figure 4:**
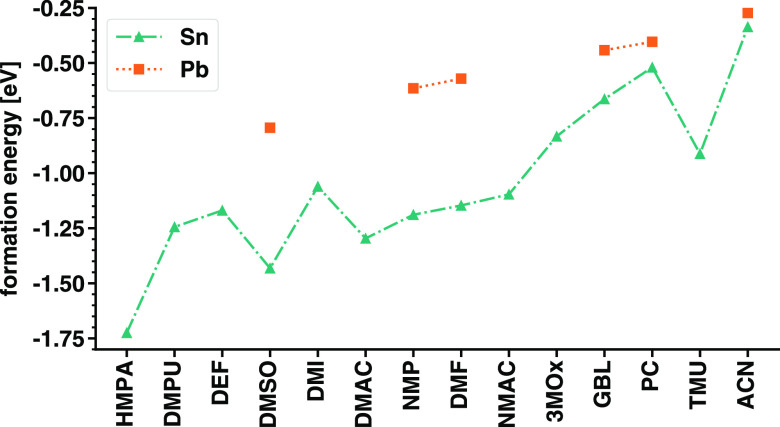
Formation energies of the 14 SnI_2_M_4_ complexes investigated in this work (green triangles); corresponding values for Pb-based counterparts taken from ref (20) (orange squares) are reported for comparison.

The results plotted in Figure 4 (data set available in Table S3) indicate that all of the considered complexes are stable. The largest formation energies are found in the presence of the HMPA solvent, which contains the P=O group. The least stable complex, on the other hand, is the one formed with ACN, the only solvent molecule included in our analysis that is bound to tin iodide through a N atom. In general, we notice a clear tendency toward decreasing stability with decreasing *D*_*N*_, in line with previous studies.^6,22,45^ Quantitative and even qualitative differences between our results and those reported in ref (6) for the same tin iodide complexes can be attributed to different definitions adopted for the formation energy as well as to the chosen computational setups.

We compare the formation energies displayed in Figure 4 with the six counterparts computed for Pb-based analogues obtained with identical computational parameters.^20^ The decreasing stability with decreasing *D*_*N*_ of the solvent discussed above is visible also in the lead-based complexes, where the trend is monotonic. The values of *E*_f_ computed for the Sn-based complexes are systematically more negative than those of the Pb-based ones,^20^ possibly on account of the smaller size of Sn compared to Pb and thus its higher tendency to form (stable) chemical bonds. In fact, in the presence of GBL and PC, whereby not all four molecules bind to SnI_2_, formation energies are very close to the corresponding values for the Pb-based counterparts. The same holds true also for SnI_2_(ACN)_4_, which exhibits a value of *E*_f_ almost identical to the one obtained in ref (20) for PbI_2_(ACN)_4_. Notice that the structural parameters of these two compounds are overlapping too (see Figure 3). In contrast, the results obtained for the tin-based complexes with DMSO, DMF, and NMP differ by several hundreds of meV from their counterparts with Pb (see Figure 4). These trends can be rationalized again with the aid of the structural analysis reported in Figure 3. In the XI_2_M_4_ complexes (with X = Sn, Pb and M = DMSO, NMP, DMF), the X–M distances are similar but the X–I separations differ substantially. This finding suggests an enhanced ionicity of the Sn–I bond compared to the Pb–I one in the presence of these high-*D*_*N*_ solvents and a consequential stabilization of the SnI_2_M_4_ complexes through the binding with solvent molecules.

### Electronic Properties

**Figure 5:**
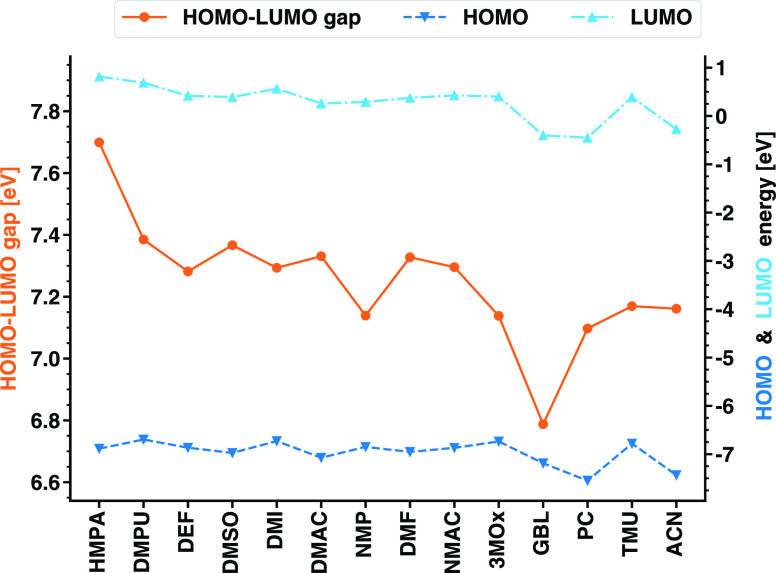
HOMO and LUMO energies of the 14 considered complexes plotted in dark and light blue, respectively, against the right *y*-axis and the resulting HOMO–LUMO gap plotted in orange against the left *y*-axis.

In the next step of our analysis, we focus on the electronic structure of the SnI_2_M_4_ complexes. We investigate the energy and the spatial distribution of the MOs, paying special attention to the highest-occupied and the lowest-unoccupied orbitals (HOMO and LUMO, respectively) and to the gap between them. The results visualized in Figure 5 (data in Table S4) suggest decreasing HOMO and LUMO energies for decreasing values of *D*_*N*_, although this trend is not monotonic. The electron-withdrawing or -donating ability of a functional group covalently bound to the edges of carbon-based molecules was shown to tune the frontier levels upward or downward with respect to a reference value.^46,47^ The involved chemistry here is different, but the above-mentioned scenario can contribute to the understanding of the trends reported in Figure 5. Overall, the values of HOMO and LUMO energies reported for all of the considered solvents vary within a window of about 1 eV, which is reflected also in their differences. The largest HOMO–LUMO gap, equal to 7.7 eV, pertains to SnI_2_(HMPA)_4_, while the smallest one (6.8 eV) pertains to SnI_2_(GBL)_4_. The gap variations in these tin-based complexes are mainly ascribed to the fluctuating HOMO energies, in contrast with the behavior of the Pb-based counterparts, where the shifts in the LUMO energies play a more crucial role.^17^

**Figure 6:**
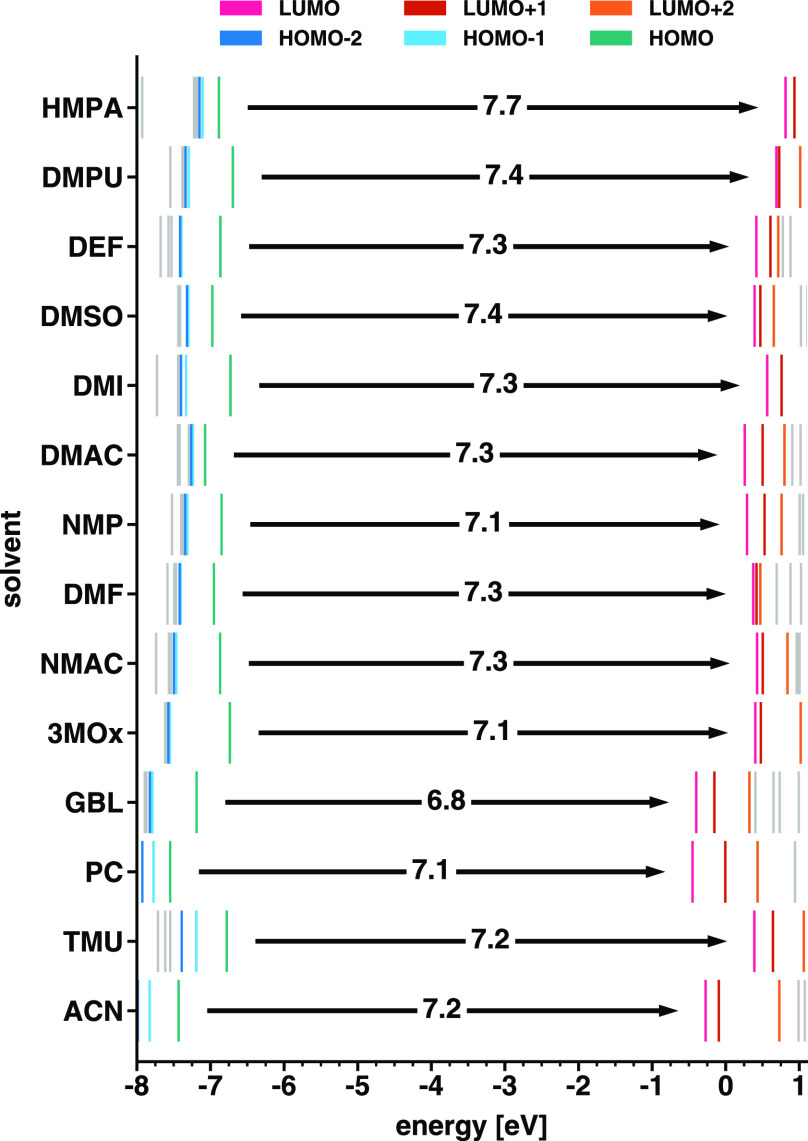
Energy distribution of the three highest-occupied and lowest-unoccupied MOs for the considered SnI_2_M_4_ complexes, with M being the solvent molecules listed on the *y*-axis. The HOMO–LUMO gaps are marked by the arrows, and their values are reported in eV.

It is worth deepening this analysis by inspecting the energy distribution of the MOs adjacent to the frontier (see Figure 6). In the majority of the considered complexes, the energetic separation between the HOMO and the HOMO–1 amounts to a few hundred meV. In contrast, the energy difference between the HOMO–1 and the HOMO–2 is much smaller, never overcoming 50 meV. A similar trend is found also for the PbI_2_M_4_ complexes.^17^ This situation is exacerbated in the tin iodide complexes with DMPU, DMI, GBL, 3MOx, and NMAC, in which the separation between the HOMO and the HOMO–1 is close to 1 eV while the energies of HOMO–1 and HOMO–2 differ by less than 50 meV. The spectrum of the unoccupied states is less regularly distributed in energy. The LUMO is generally closer to the LUMO+1 than the LUMO+1 to the LUMO+2, but no consistent trend can be identified. Yet, there are some exceptions. In the complexes solvated with DMPU, DMSO, 3MOx, and NMAC, the energy difference between the LUMO and LUMO+1 is on the order of 50 meV, while the higher unoccupied states lie at least 100 meV above the LUMO+1; in SnI_2_(NMP)_4_ and SnI_2_(PC)_4_, the LUMO, LUMO+1, and LUMO+2 are almost equally separated at intervals of 230 and 440 meV, respectively; in the presence of HMPA and DMF, the eigenvalues of the first three unoccupied states are closely packed within a range of 100 meV. It is important to underline that the orbital energies reported in Figure 6 are obtained from DFT+PCM and, as such, they cannot be interpreted as excitation energies in the framework of Koopman’s theorem.^48^ They only inform us about the distribution of the single-particle states in the electronic structure of the complexes, thus providing a useful starting point for the analysis of the optical properties below.

**Figure 7:**
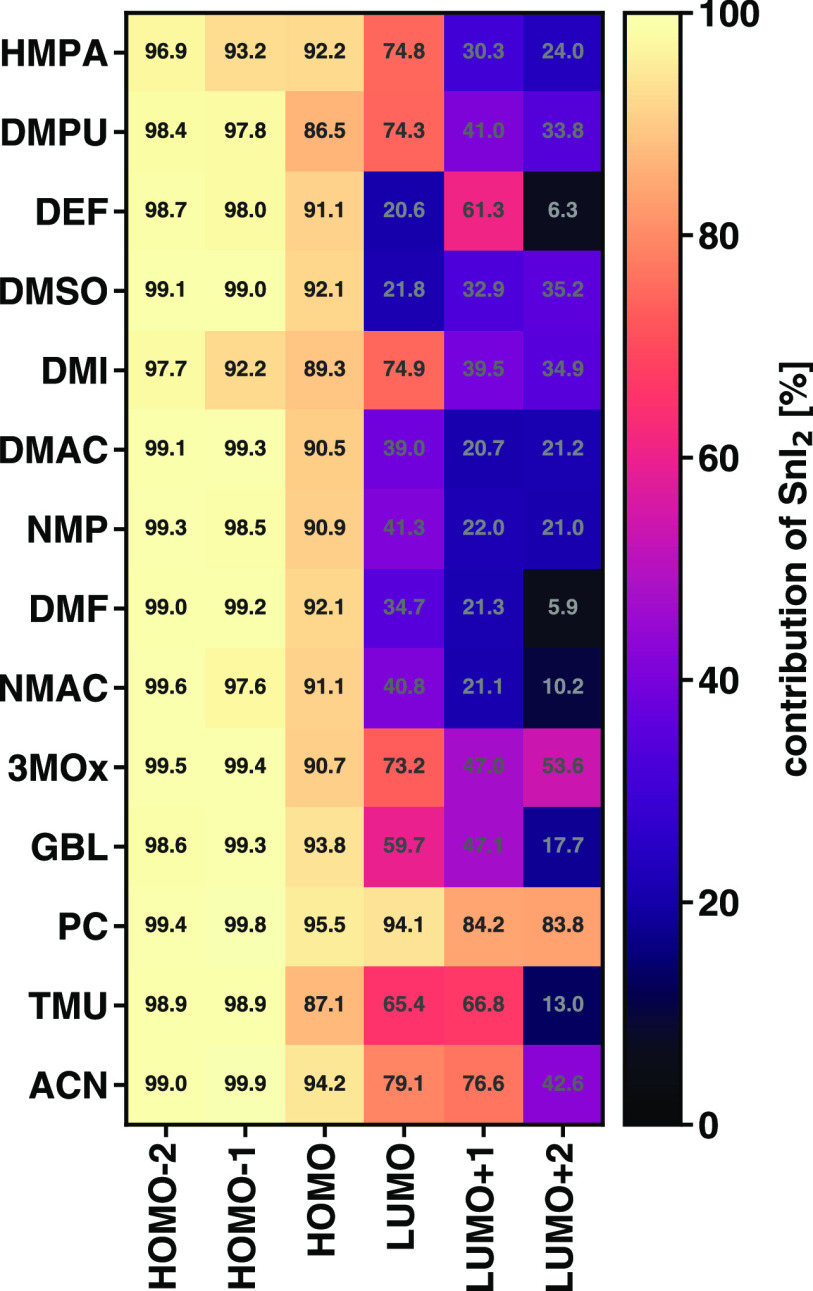
SnI_2_ contributions to the indicated MOs calculated with the NAO method.

To complement the analysis of these energetic trends, we examine the spatial distribution of the MOs within the complexes. For quantitative analysis, we make use of the NAO method and evaluate the SnI_2_ contribution to a certain orbital as the sum of the corresponding charge density on the Sn and I atoms (see Figure 7). The considered occupied states (HOMO–2, HOMO–1, and HOMO) are almost entirely localized on SnI_2_. In the HOMO–1 and HOMO–2, less than 10% of the wave function is localized on the solvent, while in the HOMO, it is below 15%. In the unoccupied region, the situation changes and substantial differences can be seen depending on the solvent. However, in this case, no clear trends can be identified according to the donor number. In the SnI_2_(PC)_4_ complex, the LUMO is localized by more than 94% on the tin iodide center. The presence of HMPA, DMPU, DMI, 3MOx, and ACN triggers a similar behavior with about 75% of the orbital distributed on SnI_2_. In SnI_2_(GBL)_4_ and SnI_2_(TMU)_4_, approximately 60 and 65% of the LUMO, respectively, sit on the metal halide backbone. In all other considered systems, the larger portion of the wave function of the lowest-unoccupied state is on the solvent molecules (see Figure 7). At higher energies, the solvent contributions become even more prominent, with the exception of the SnI_2_(PC)_4_ complex where all MOs analyzed in Figure 7 are mainly focused on the SnI_2_ unit. Earlier results obtained for equivalent PbI_2_M_4_ systems indicate a similar trend, with the frontier states localized on the PbI_2_ kernel with negligible or even vanishing contributions from the surrounding solvent molecules.^17,20^ Further details on the orbital energies and spatial distributions of the inspected SnI_2_M_4_ complexes can be found in the Supporting Information, Figures S1–S6.

### Optical Properties

**Figure 8:**
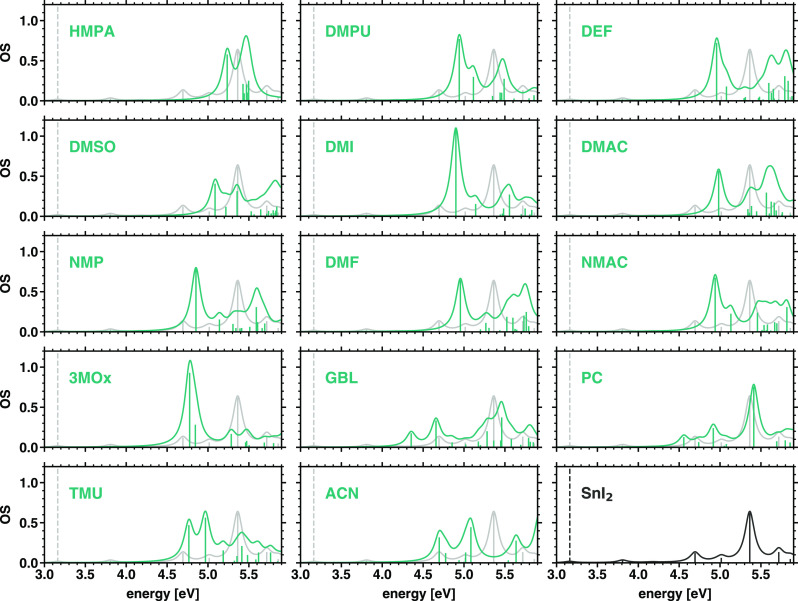
Optical absorption spectra of the 14 considered SnI_2_M_4_ complexes and of the SnI_2_ molecule in an implicit DMSO solution for reference. The absorption spectrum was calculated with a Lorentzian broadening of 70 meV.

The absorption spectra of the SnI_2_M_4_ complexes are reported in Figure 8, where the result obtained for the isolated SnI_2_ molecule in an implicit DMSO solution is displayed for comparison on the bottom-right panel. We checked that the choice of the implicit solvent in this reference calculation does not impact the result obtained for the spectrum, as expected from previous analysis on other solvated compounds.^48^ The quantum-mechanical interactions with solvent molecules in the SnI_2_M_4_ compounds increase the energy of the first excited state from 3.2 eV (dashed bar in Figure 8) to 4 eV or higher (first peak in the green curves), overall redistributing the spectral weight toward lower energies. In fact, in the spectrum of SnI_2_, the lowest-energy excitations have very weak intensity: the first peaks distinguishable in Figure 8, bottom-right panel, are above 4.5 eV, and the most intense excitation in the displayed window is close to 5.4 eV. Yet, weak but nonzero resonances are present at 3.1 and 3.8 eV. In the majority of the spectra of the considered SnI_2_M_4_ complexes, on the other hand, the most intense resonance in the considered energy range is found below 5 eV. Exceptions are the spectrum of SnI_2_(ACN)_4_, which is dominated by two maxima of almost equal intensity at 4.7 and 5.1 eV, and the results obtained for SnI_2_(DMSO)_4_ and SnI_2_(HMPA)_4_, where the first bright transition is well above 5 eV. Notice that in the last two compounds the HOMO–LUMO gap is the largest, with values of 7.7 eV [SnI_2_(HMPA)_4_] and 7.4 eV [SnI_2_(DMSO)_4_]; see Figure 6. The spectrum of SnI_2_(PC)_4_ is even more peculiar: it exhibits a remarkable similarity with the one of SnI_2_ alone. This finding can be readily understood by recalling that both the HOMO and the LUMO in this solution complex are predominantly localized on the tin iodide center with negligible wave function delocalization on the solvent molecules (see Figure 7). A similar analysis performed on the subsequent four excitations (second to fifth excited states) in the spectrum of the complexes reveals a similar trend in terms of energy vs donor number. However, it should be mentioned that these higher-lying excitations are dark in all systems with only a couple of exceptions (see Figure S7). This characteristic is due to the minimized wave function overlap between the states involved in the corresponding transitions (see Figures S8–S12), which occur between orbitals away from the frontier and, as such, are localized on the different groups constituting the complexes (see Figure 7).

**Figure 9:**
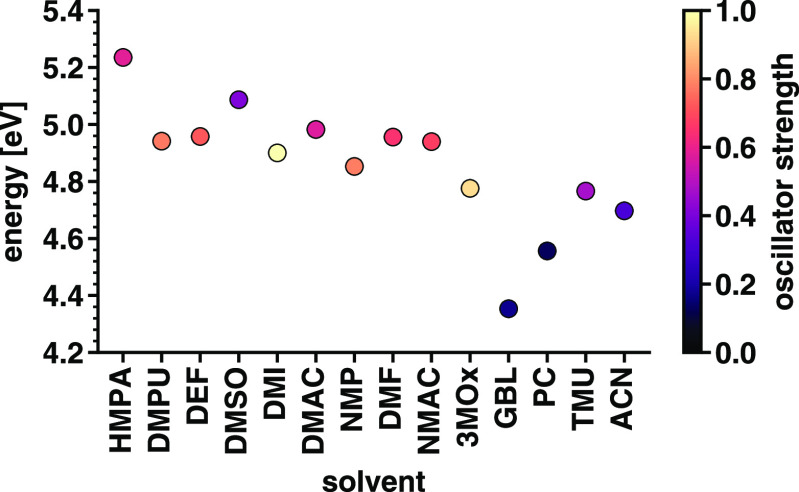
Energy and oscillator strength of the first excitation calculated for the 14 considered complexes. The solvent molecules on the *x*-axis are displayed with a decreasing donor number from left to right.

The characteristics of the first excitations in all considered complexes are summarized in Figure 9 (see also Table S5). In the graph correlating the energy and oscillator strength (OS) of the lowest-energy transition, we notice some trends. First, there is a consistent (although not monotonic) red shift of the first excitation with decreasing values of *D*_*N*_, which is in general agreement with the measured UV–visible spectra of SnI_2_ solutions.^45,49^ Moreover, in accordance with physical intuition, the energy of the lowest-energy excitation correlates with the HOMO–LUMO gap. Similar to the spectral weight, also this quantity decreases for decreasing values of *D*_*N*_; moreover, the notable exception of SnI_2_(GBL)_4_ can be noticed as well in the trend of the HOMO–LUMO gaps in Figure 5.

**Figure 10:**
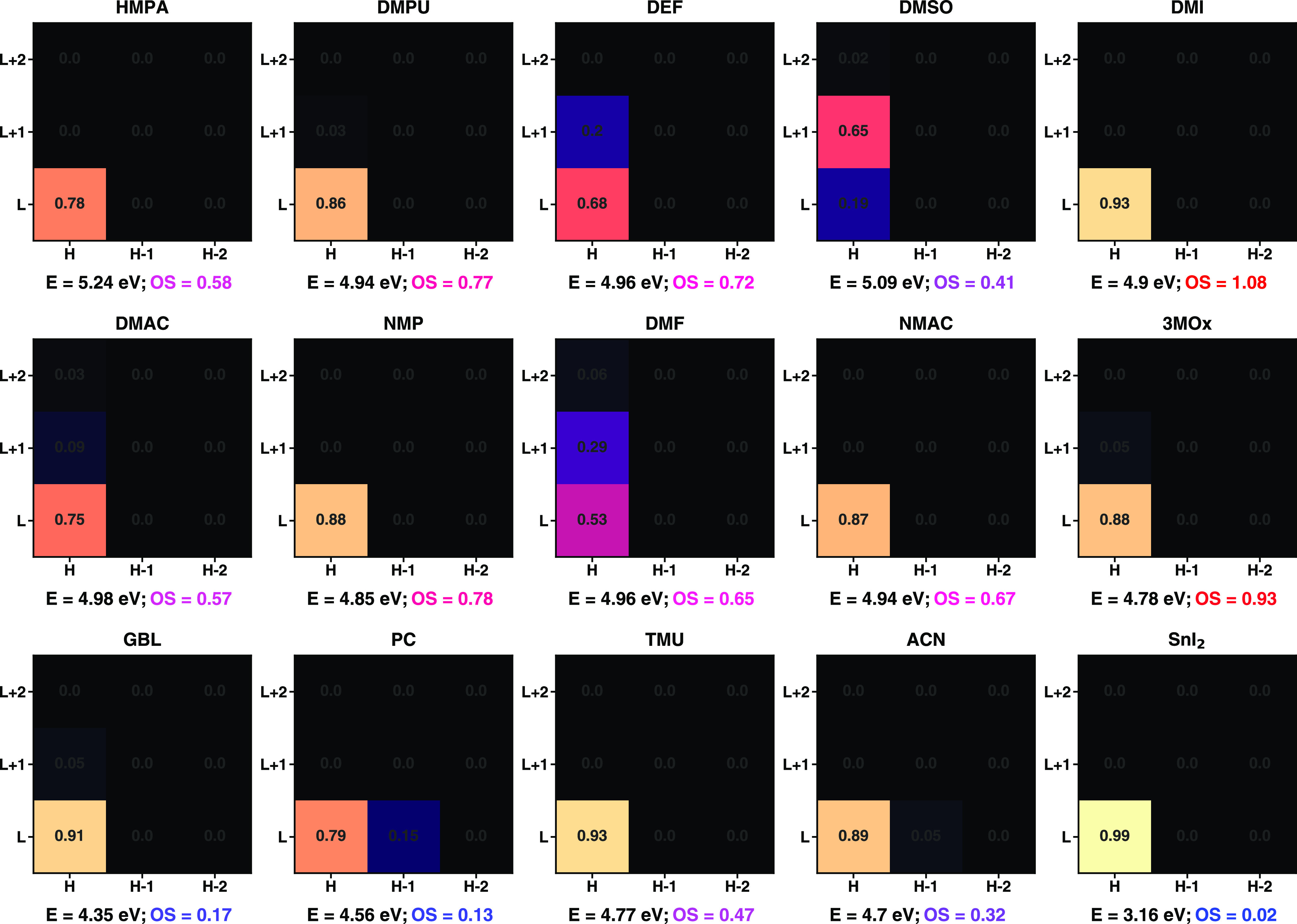
Composition of the first excited state of the 14 SnI_2_M_4_ complexes indicated by their solvent molecule M and of the SnI_2_ molecule calculated in an implicit DMSO solution. The contribution ∈ [0, 1] of the transition from the occupied (*x*-axis) to the unoccupied (*y*-axis) state is displayed in the corresponding grid square. H stands for HOMO and L for LUMO. For each transition, the energy (*E*) and oscillator strength (OS) are reported.

The low-energy optical transitions occurring in the complexes including solvents with high donor numbers are generally more intense than those in the spectra of low-*D*_*N*_ solvents. This feature can be related to the orbital distribution reported in Figure 7 through the analysis of the excitations in terms of single-particle transitions (see Figure 10); in each box, the relative amount ∈ [0, 1] of each orbital transition is displayed together with the excitation energy and OS. Not unexpectedly, the first excitation of almost all complexes stems primarily from the HOMO → LUMO transition. As such, the trend obtained for the HOMO–LUMO gaps (Figure 5) is generally reflected in the first excitation energy (Figure 9), as anticipated above. An exception is given by SnI_2_(DMSO)_4_, where the main contribution comes from a transition from the HOMO to the LUMO+1. A likely reason for the atypical result, which corresponds to comparably lower measured absorption of Sn perovskite films with DMSO compared to other solvents,^45^ can be related to the larger difference between the spatial distribution of the HOMO and LUMO in SnI_2_(DMSO)_4_. While, like in all complexes, the HOMO is almost entirely formed by s- and p-states of Sn and I atoms (see Figures S3 and S6), the wave function of the LUMO is mainly distributed on the solvent atoms. This hypothesis is supported by the fact the two other complexes with lower HOMO → LUMO contribution to the first excitation, namely, SnI_2_(DEF)_4_ and SnI_2_(DMF)_4_, have also very low SnI_2_ contributions to the LUMO with 20.6 and 34.7%, respectively (see Figure 7). However, contrary to the results obtained for the PbI_2_M_4_ complexes,^17^ a low HOMO → LUMO contribution does not necessarily correspond to a low OS of the first excitation in the SnI_2_M_4_ systems. In fact, while the interaction with solvent molecules causes a significant reduction of the OS due to charge delocalization away from the PbI_2_ unit in the Pb-based complexes, this is not the case in the Sn-based systems.

## Summary and Conclusions

In summary, we have investigated the structural, energetic, electronic, and optical properties of 14 tin iodide solution complexes. As model systems, we have considered complexes with formula SnI_2_M_4_, where M are common solvents with complementary chemical characteristics and varying donor numbers. With this approach, we were able to model the short-range quantum-mechanical interactions between solute and solvents that are expected to dominate the electronic and optical response of the systems. All the investigated structures are stable, although, in two compounds, not all solvent molecules form chemical bonds with SnI_2_. The formation energy, introduced as a metric for stability, decreases in magnitude with decreasing values of *D*_*N*_, suggesting that the ability of the solvents to bind to tin iodide depends directly on their effectiveness in donating electrons. The orbital energies do not follow an equally clear trend, although solvents with higher donor numbers tend to have higher energies for the frontier orbitals and also for the gap between them. The energetic distribution of the highest occupied and lowest unoccupied states could not be straightforwardly related to *D*_*N*_ either, although their spatial distribution follows a clear trend. The occupied orbitals are predominantly localized on the SnI_2_ unit while the unoccupied ones are, in most cases, largely distributed also on the solvent molecules. The limited wave function overlap between the frontier states is responsible for the low OS of the first excitation in most complexes. This being said, the presence of the coordinated solvent molecules red shifts the spectral weight in comparison to the result obtained for SnI_2_ alone.

To conclude, our results provide a comprehensive characterization of the fundamental properties of tin iodide solution complexes. Thanks to the fully quantum-mechanical approach adopted in this study and the deep level of our analysis, our findings complement the existing knowledge available for these systems, offering a robust reference for data analysis, interpretation, and understanding. Moreover, they can be the basis for future investigations on halide perovskite solution precursors and/or compounds produced in subsequent synthesis steps of tin halide perovskite thin films.

## Data Availability

The data that support the findings of this study are openly available in Zenodo at 10.5281/zenodo.7729359.

## Supporting Information Available

Additional details on the ab initio calculation of Gutmann’s donor number^38,50,51^ together with the data related to structural properties, orbital energies, and spatial distribution, and analysis of the optical excitations (PDF)
