# Correction to “Electronic Structure and Optical Properties of Tin Iodide Solution Complexes”

**DOI:** 10.1021/acs.jpca.3c05058

**Published:** 2023-08-17

**Authors:** Freerk Schütt, Ana M. Valencia, Caterina Cocchi

In our recent publication, we plotted Figure 1 with an unintentional mistake in the reported value for the donor number (*D*_*N*_) of the solvent tetramethylurea (TMU), which can be found in Table S1 of the Supporting Information. The correct version of Figure 1 is displayed below.

**Figure 1:**
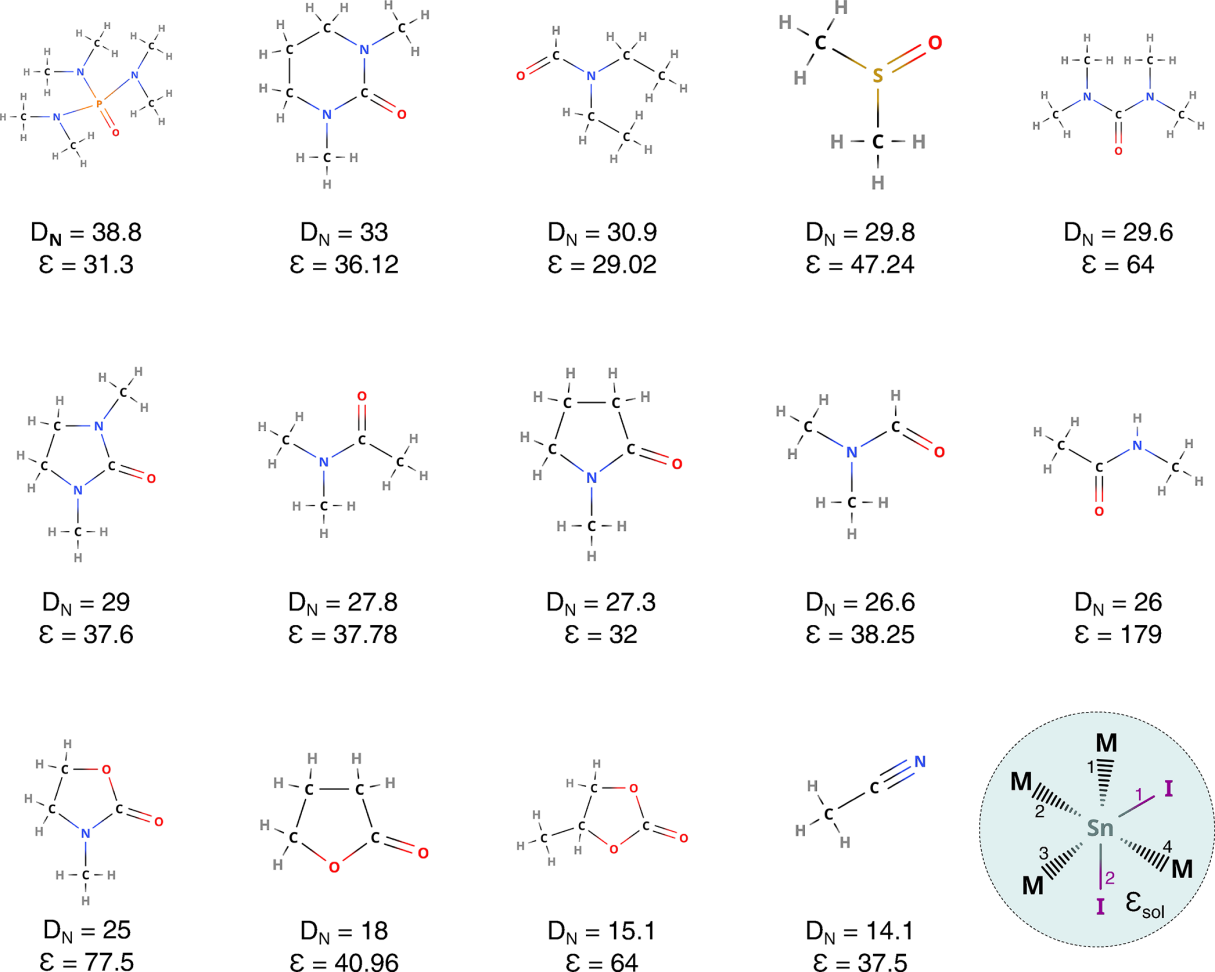
Overview of the 14 solvents considered in this study with their respective Gutmann’s donor number (*D*_*N*_) and static dielectric constant (ε): hexamethylphosphoramide (HMPA), *N*,*N*′-dimethylpropyleneurea (DMPU), *N*,*N*-diethylformamide (DEF), dimethyl sulfoxide (DMSO), tetramethylurea (TMU), 1,3-dimethyl-2-imidazolidinone (DMI), dimethylacetamide (DMAC), *N*-methyl-2-pyrrolidone (NMP), dimethylformamide (DMF), *N*-methylacetamide (NMAC), 3-methyl-2-oxazolidinone (3MOx), γ-butyrolactone (GBL), propylene carbonate (PC), and acetonitrile (ACN). On the bottom right panel, a sketch of the SnI_2_M_4_ compounds in the solvent cavity is reported: the numbers label the Sn-I (purple) and the Sn-M (black) bonds.

