# Supplementary material for: Electronic Structure and Optical Properties of Tin Iodide Solution Complexes
Source: J Phys Chem A. 2023 May 12;127(20):4463–72. doi: 10.1021/acs.jpca.3c01754 (PMC10226128; doi:10.1021/acs.jpca.3c01754)
Supplement: Supplementary file 1 — jp3c01754_si_001.pdf [file jp3c01754_si_001.pdf]

Supporting Information: Electronic Structure and Optical Properties of Tin Iodide Solution Complexes

Freerk Schütt,† Ana M. Valencia,†,¶ and Caterina Cocchi*,†,‡,¶

†*Carl-von-Ossietzky Universität Oldenburg, Institute of Physics, 26129 Oldenburg, Germany*

‡*Carl-von-Ossietzky Universität Oldenburg, Center for Nanoscale Dynamics, 26129 Oldenburg, Germany*

¶*Humboldt-Universität zu Berlin, Physics Department and IRIS Adlershof, 12489 Berlin, Germany*

E-mail: caterina.cocchi@uni-oldenburg.de

Supporting Information Available

Calculation of Donor Number

The donor number D_N of a solvent S is defined as the negative enthalpy change

$$D_N = -\Delta H_{D_N}, \tag{1}$$

usually expressed in kcal/mol, for a 1:1 adduct formation between the solvent S and the reference Lewis acid antimony pentachloride $SbCl_5$ at room temperature in the inert solvent 1,2-Dichloroethane (DCE).[S1] The simplified solvation process can be schematically described by the chemical reaction

$$\mathrm{S} \cdot \mathrm{DCE} + \mathrm{DCE} \cdot (\mathrm{SBCl_5}) \rightleftharpoons (\mathrm{S} \cdot (\mathrm{SbCl_5})) \cdot \mathrm{DCE} + \mathrm{DCE}, \tag{2}$$

with the enthalpy change ΔH_{D_N}.[S2,S3] Under the approximation that enthalpy changes are equivalent to reaction energy changes, ΔH_{D_N} can be calculated from the ionization potential (IP) and electron affinity (EA) of the molecules involved.

Following the method proposed in Ref. S2, we calculated the IP and EA of the inert DCE, the reference Lewis acid $SBCl_5$, and a selection of 11 reference solvents, including 3MOx and NMAC. For these calculations, the same functional and basis sets were used as for the single point calculations described above. The resulting values for the IP and EA are listed in Table S1, together with the experimentally determined reference values and the calculated values for the donor numbers. The final values of D_N were calculated using the perturbed implicit solvation approach described in Ref. S2 with the provided Python script.[S4] Overall, the theoretically determined values show acceptable agreement with the experimental results, giving donor numbers for 3MOx and NMAC of $D_{N,\mathrm{3MOx}} = 25.1$ and $D_{N,\mathrm{NMAC}} = 26$.

Table S1: Ionization potential (IP), electron affinity (IP), experimentally and theoretically determined donor number, $D_{N,exp}$ and $D_{N,theo}$, respectively, of the eleven solvents, including 3MOx and NMAC.

	IP [eV]	EA [eV]	$D_{N,exp}$ [kcal/mol]	$D_{N,theo}$ [kcal/mol]
DCE	9.02	0.23		
$SbCl_5$	8.54	5.48		
3MOx	6.73	0.64		25.1
DEF	6.69	-0.94	30.9	31
DMAC	6.98	-0.14	27.8	26
DMF	6.74	-0.87	26.6	30.4
DMI	6.1	0.26	29	31.8
DMPU	6.13	0.46	33	30.7
GBL	7.73	0.94	18	17.9
HMPA	5.87	-0.15	38.8	35.5
NMAC	6.98	-0.14		26
PC	8.32	1.04	15.1	14.8
TMU	6.1	0.54	29.6	30.7

Structural Properties

Table S2: Structural properties of the 14 complexes, including the distances between the Sn and two I atoms (Sn-I distances), the angle within the SnI_2 molecule (I-Sn-I angle), and the distances between the Sn atom and the four solvent molecules (Sn-M distances).

	Sn-I distances [Å]			Sn-M distances [Å]			
	1	2	I-Sn-I [°]	1	2	3	4
SnI_2	2.90	2.89	98.99				
$SnI_2(HMPA)_4$	4.32	4.23	98.47	2.39	2.36	2.15	2.14
$SnI_2(DMPU)_4$	3.72	3.67	103.53	2.65	2.28	2.26	2.24
$SnI_2(DEF)_4$	4.01	3.45	131.21	2.42	2.33	2.28	2.24
$SnI_2(DMSO)_4$	4.26	3.81	124.53	2.41	2.23	2.19	2.18
$SnI_2(DMI)_4$	3.62	3.57	119.80	2.52	2.46	2.24	2.23
$SnI_2(DMAC)_4$	4.89	3.61	132.18	2.41	2.32	2.22	2.18
$SnI_2(NMP)_4$	3.97	3.69	119.60	2.42	2.36	2.22	2.19
$SnI_2(DMF)_4$	4.10	3.54	110.91	2.38	2.36	2.27	2.23
$SnI_2(NMAC)_4$	3.78	3.54	117.51	2.45	2.39	2.25	2.25
$SnI_2(3\,MOx)_4$	3.46	3.40	120.33	2.53	2.41	2.37	2.32
$SnI_2(GBL)_4$	3.34	3.32	112.14	3.86	2.37	2.37	2.23
$SnI_2(PC)_4$	3.18	2.99	93.10	4.38	3.52	2.50	2.28
$SnI_2(TMU)_4$	3.44	3.35	88.54	2.58	2.58	2.46	2.27
$SnI_2(ACN)_4$	3.06	3.04	96.96	2.94	2.83	2.82	2.44

Stability

Table S3: Formation energy of the SnI_2M_4 complexes ($E_{f,\mathrm{Sn}}$) and of the six PbI_2M_4 complexes ($E_{f,\mathrm{Pb}}$) from Ref. S5 calculated at the same level of theory.

	$E_{f,\mathrm{Sn}}$ [eV]	$E_{f,\mathrm{Pb}}$ [eV]
HMPA	-1.72	
DMPU	-1.24	
DEF	-1.17	
DMSO	-1.43	-0.794
DMI	-1.06	
DMAC	-1.30	
NMP	-1.19	-0.615
DMF	-1.15	-0.571
NMAC	-1.10	
3MOx	-0.83	
GBL	-0.66	-0.442
PC	-0.52	-0.404
TMU	-0.91	
ACN	-0.34	-0.273

Electronic Properties

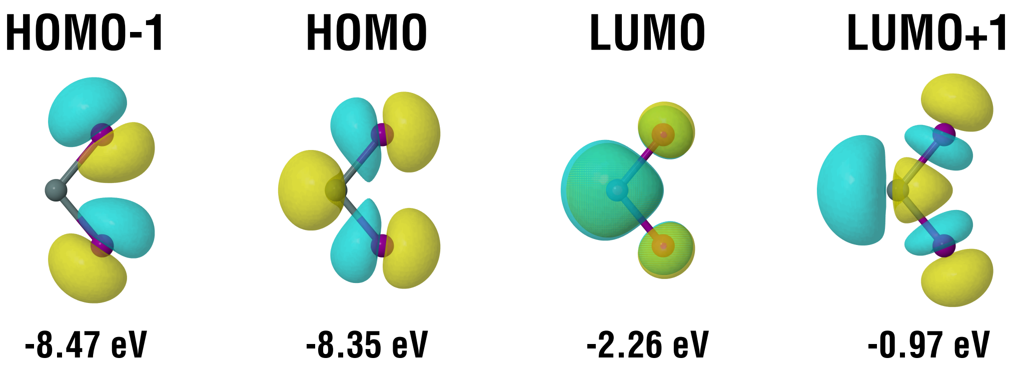

Figure S1: Energy and spatial distribution of selected molecular orbitals of the SnI_2 molecule.

Table S4: Energy eigenvalues of the HOMO and the LUMO, and HOMO-LUMO gap for the 14 considered SnI_2M_4 complexes and for the SnI_2 in an implicit DMSO solution.

	HOMO [eV]	LUMO [eV]	HOMO-LUMO gap [eV]
SnI_2	-8.35	-2.26	6.09
$SnI_2(HMPA)_4$	-6.89	0.81	7.70
$SnI_2(DMPU)_4$	-6.70	0.69	7.38
$SnI_2(DEF)_4$	-6.87	0.42	7.28
$SnI_2(DMSO)_4$	-6.97	0.39	7.37
$SnI_2(DMI)_4$	-6.73	0.56	7.29
$SnI_2(DMAC)_4$	-7.07	0.26	7.33
$SnI_2(NMP)_4$	-6.85	0.29	7.14
$SnI_2(DMF)_4$	-6.95	0.37	7.33
$SnI_2(NMAC)_4$	-6.87	0.43	7.30
$SnI_2(3\,MOx)_4$	-6.74	0.40	7.14
$SnI_2(GBL)_4$	-7.19	-0.40	6.79
$SnI_2(PC)_4$	-7.55	-0.45	7.10
$SnI_2(TMU)_4$	-6.78	0.39	7.17
$SnI_2(ACN)_4$	-7.43	-0.27	7.16

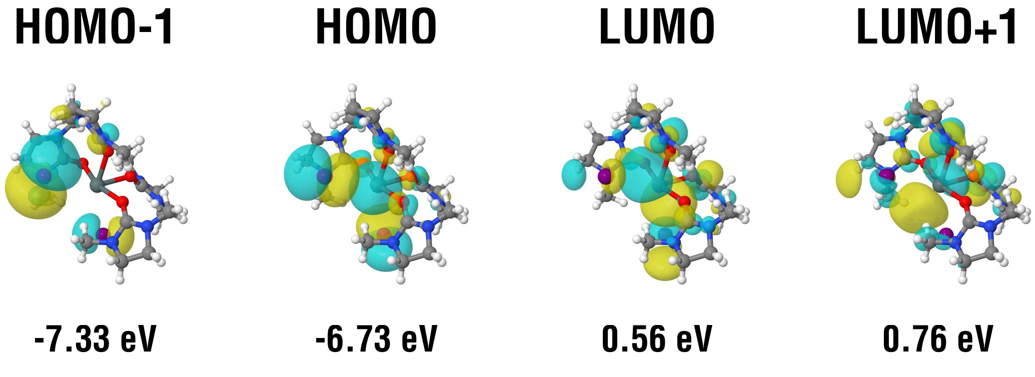

Figure S2: Energy and spatial distribution of selected molecular orbitals of the $SnI_2(DMI)_4$ complex.

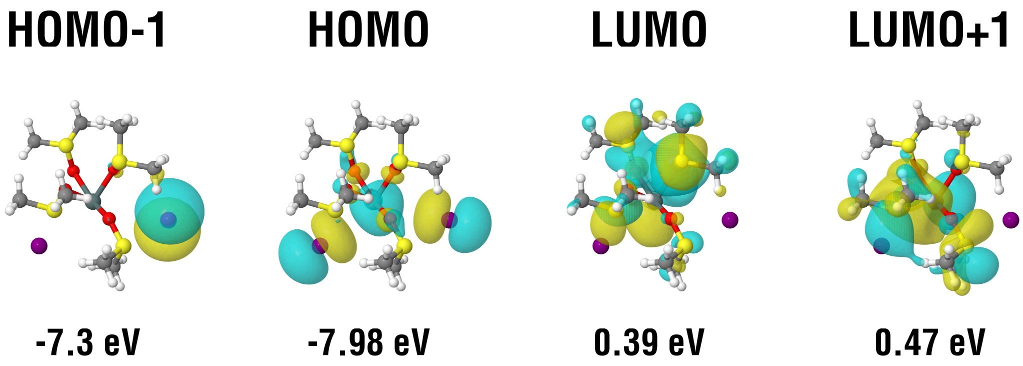

Figure S3: Energy and spatial distribution of selected molecular orbitals of the $SnI_2(DMSO)_4$ complex.

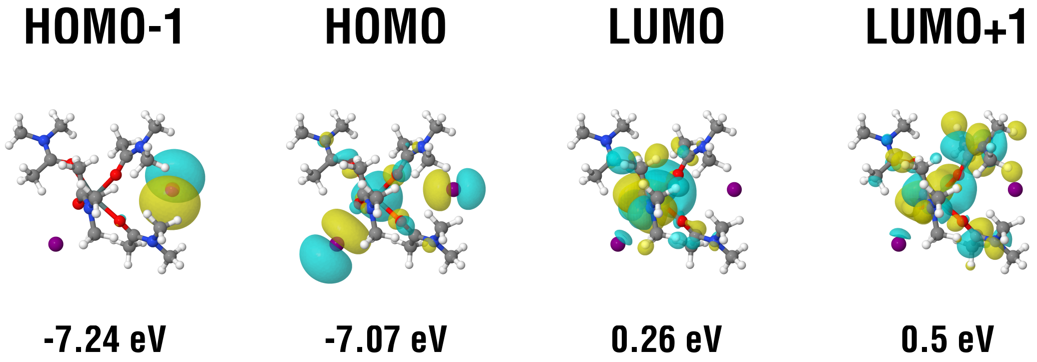

Figure S4: Energy and spatial distribution of selected molecular orbitals of the $SnI_2(DMAC)_4$ complex.

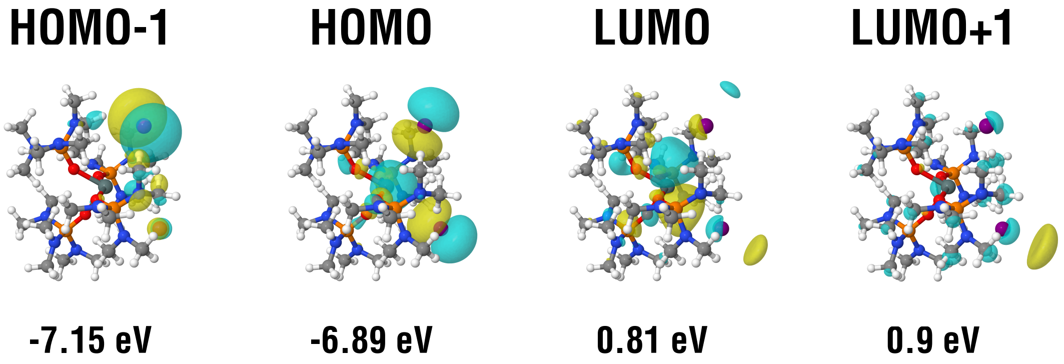

Figure S5: Energy and spatial distribution of selected molecular orbitals of the $SnI_2(HMPA)_4$ complex.

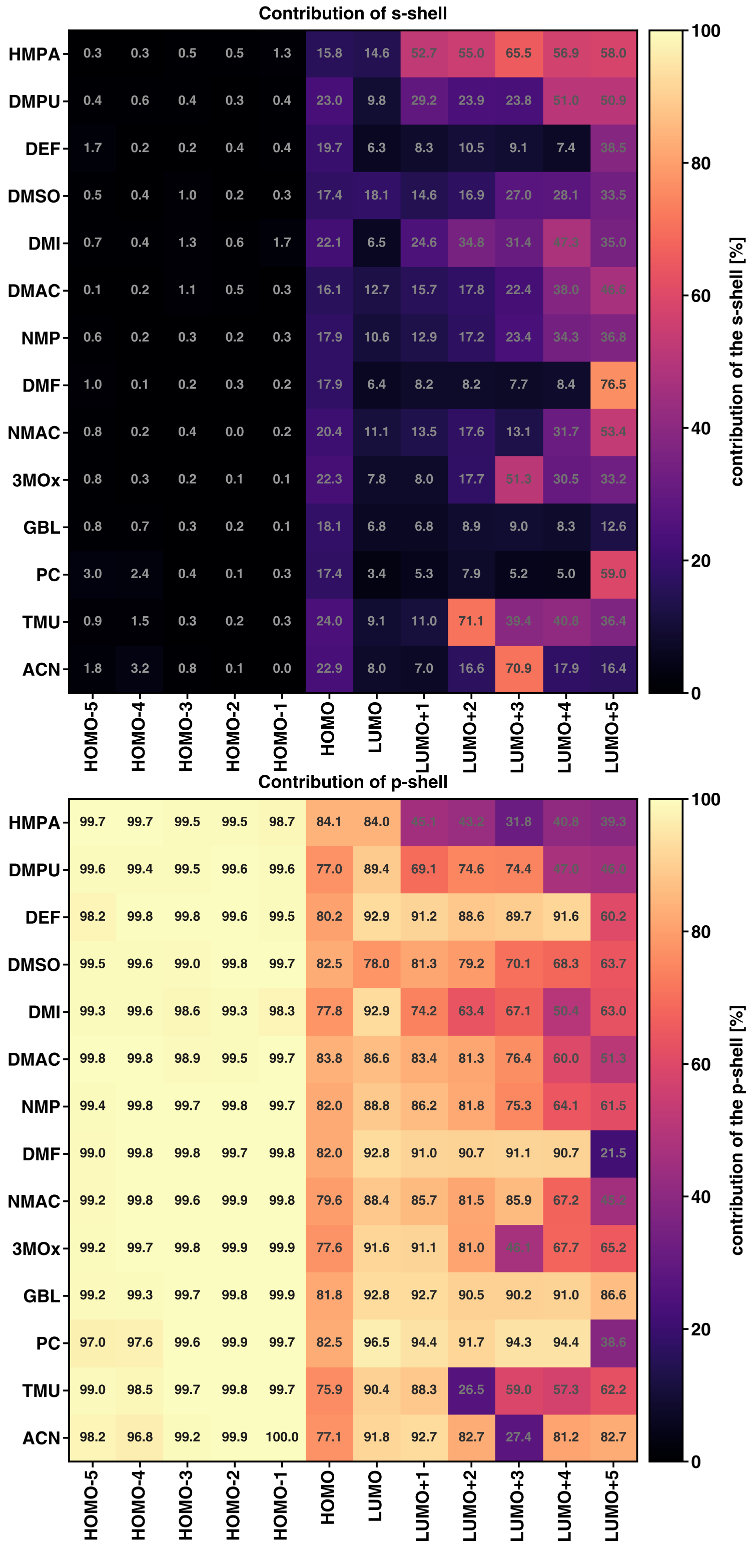

Figure S6: Contributions of the s-shell (top) and p-shell (bottom) to the molecular orbitals of the 14 SnI_2M_4 complexes.

Optical Properties

Table S5: Energy, oscillator strength, and dominant electronic transitions of the first optical excitation of the 14 $\mathbf{SnI_2M_4}$ complexes and of the $\mathbf{SnI_2}$ molecule.

	energy [eV]	OS	composition
SnI_2	3.16	0.02	HOMO → LUMO (99%); H-4 → LUMO (2%);
$SnI_2(HMPA)_4$	5.24	0.58	HOMO → LUMO (78%); H-5 → L+2 (3%);
$SnI_2(DMPU)_4$	4.94	0.77	HOMO → LUMO (86%); H-5 → L+1 (3%);
$SnI_2(DEF)_4$	4.96	0.72	HOMO → LUMO (68%); HOMO → L+4 (2%);
$SnI_2(DMSO)_4$	5.09	0.41	HOMO → L+1 (65%); HOMO → LUMO (19%);
$SnI_2(DMI)_4$	4.90	1.08	HOMO → LUMO (93%);
$SnI_2(DMAC)_4$	4.98	0.57	HOMO → LUMO (75%); HOMO → L+1 (9%);
$SnI_2(NMP)_4$	4.85	0.78	HOMO → LUMO (88%);
$SnI_2(DMF)_4$	4.96	0.65	HOMO → LUMO (53%); HOMO → L+4 (5%);
$SnI_2(NMAC)_4$	4.94	0.67	HOMO → LUMO (87%); HOMO → L+3 (2%);
$SnI_2(3\,MOx)_4$	4.78	0.93	HOMO → LUMO (88%); HOMO → L+1 (5%);
$SnI_2(GBL)_4$	4.35	0.17	HOMO → LUMO (91%); HOMO → L+1 (5%);
$SnI_2(PC)_4$	4.56	0.13	HOMO → LUMO (79%); H-1 → LUMO (15%);
$SnI_2(TMU)_4$	4.77	0.47	HOMO → LUMO (93%);
$SnI_2(ACN)_4$	4.70	0.32	HOMO → LUMO (89%); H-1 → LUMO (5%);

Table S6: Energy, oscillator strength, and dominant electronic transitions of the second optical excitation of the 14 SnI_2M_4 complexes and of the SnI_2 molecule.

	energy [eV]	OS	composition
SnI_2	3.46	0.00	H-1 → LUMO (100%); H-1 → L+11 (0%);
$SnI_2(HMPA)_4$	5.43	0.21	HOMO → L+1 (33%); H-1 → LUMO (6%);
$SnI_2(DMPU)_4$	5.12	0.30	HOMO → L+1 (73%); HOMO → LUMO (3%);
$SnI_2(DEF)_4$	5.08	0.18	HOMO → L+1 (68%); HOMO → LUMO (18%);
$SnI_2(DMSO)_4$	5.22	0.12	HOMO → LUMO (57%); H-6 → LUMO (3%);
$SnI_2(DMI)_4$	5.14	0.15	HOMO → L+1 (85%);
$SnI_2(DMAC)_4$	5.34	0.09	HOMO → L+1 (44%); H-4 → LUMO (19%);
$SnI_2(NMP)_4$	5.14	0.15	HOMO → L+1 (82%); H-6 → L+1 (3%);
$SnI_2(DMF)_4$	5.20	0.03	HOMO → L+2 (68%); HOMO → LUMO (10%);
$SnI_2(NMAC)_4$	5.13	0.23	HOMO → L+1 (83%); H-6 → L+1 (4%);
$SnI_2(3\,MOx)_4$	4.85	0.28	HOMO → L+1 (88%); HOMO → LUMO (5%);
$SnI_2(GBL)_4$	4.66	0.33	HOMO → L+1 (84%); HOMO → LUMO (5%);
$SnI_2(PC)_4$	4.74	0.07	H-1 → LUMO (67%); H-4 → LUMO (9%);
$SnI_2(TMU)_4$	4.97	0.56	HOMO → L+1 (88%); H-1 → LUMO (2%);
$SnI_2(ACN)_4$	4.78	0.12	HOMO → L+1 (53%); H-1 → LUMO (36%);

Table S7: Energy, oscillator strength, and dominant electronic transitions of the third optical excitation of the 14 SnI_2M_4 complexes and of the SnI_2 molecule.

	energy [eV]	OS	composition
SnI_2	3.81	0.03	H-2 → LUMO (95%); H-1 → L+1 (2%);
$SnI_2(HMPA)_4$	5.44	0.09	H-1 → LUMO (31%); H-1 → L+2 (14%);
$SnI_2(DMPU)_4$	5.35	0.06	HOMO → L+2 (56%); H-3 → L+1 (5%);
$SnI_2(DEF)_4$	5.29	0.03	HOMO → L+3 (51%); H-6 → L+3 (3%);
$SnI_2(DMSO)_4$	5.36	0.31	HOMO → L+2 (72%); HOMO → LUMO (8%);
$SnI_2(DMI)_4$	5.44	0.02	H-1 → LUMO (46%); HOMO → L+5 (2%);
$SnI_2(DMAC)_4$	5.36	0.04	H-4 → LUMO (44%); H-4 → L+1 (10%);
$SnI_2(NMP)_4$	5.31	0.10	HOMO → L+2 (50%); H-1 → LUMO (17%);
$SnI_2(DMF)_4$	5.27	0.11	HOMO → L+3 (80%); HOMO → L+4 (2%);
$SnI_2(NMAC)_4$	5.46	0.24	HOMO → L+2 (31%); HOMO → L+4 (21%);
$SnI_2(3\,MOx)_4$	5.28	0.17	HOMO → L+2 (73%); HOMO → L+9 (4%);
$SnI_2(GBL)_4$	4.82	0.02	H-1 → LUMO (47%); H-4 → LUMO (3%);
$SnI_2(PC)_4$	4.90	0.01	H-3 → LUMO (52%); H-4 → LUMO (2%);
$SnI_2(TMU)_4$	5.19	0.16	H-1 → LUMO (79%); H-4 → LUMO (11%);
$SnI_2(ACN)_4$	4.94	0.03	H-2 → LUMO (39%); HOMO → L+1 (3%);

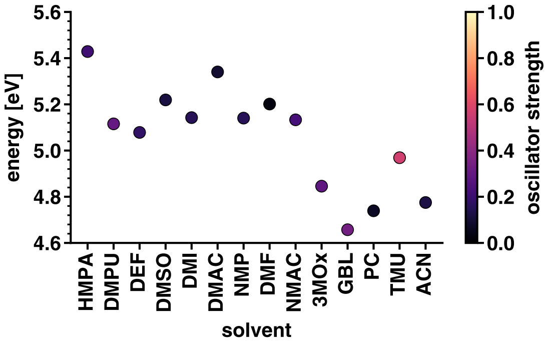

(a) 2nd excitation

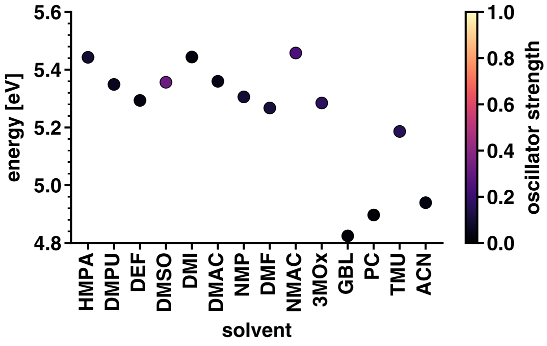

(b) 3rd excitation

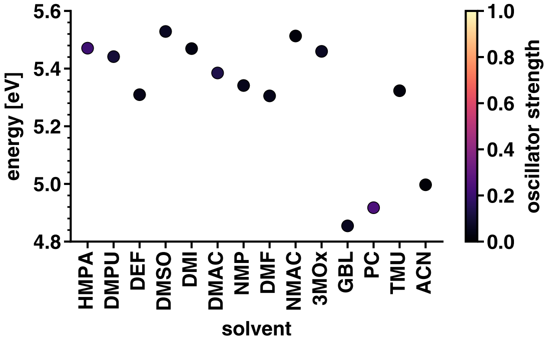

(c) 4th excitation

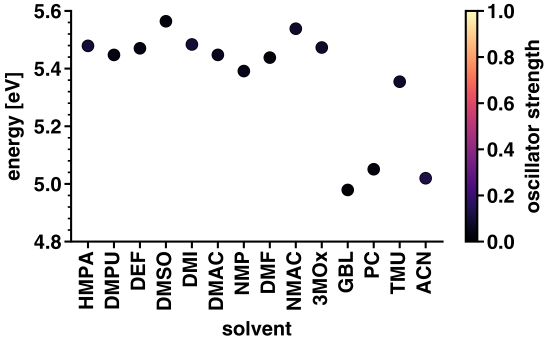

(d) 5th excitation

Figure S7: Energy and oscillator strength of the (a) second, (b) third, (c) fourth, and (d) fifth optical excitation in the spectra calculated for the 14 complexes. The solvents on the x-axes are displayed with decreasing donor numbers from left to right.

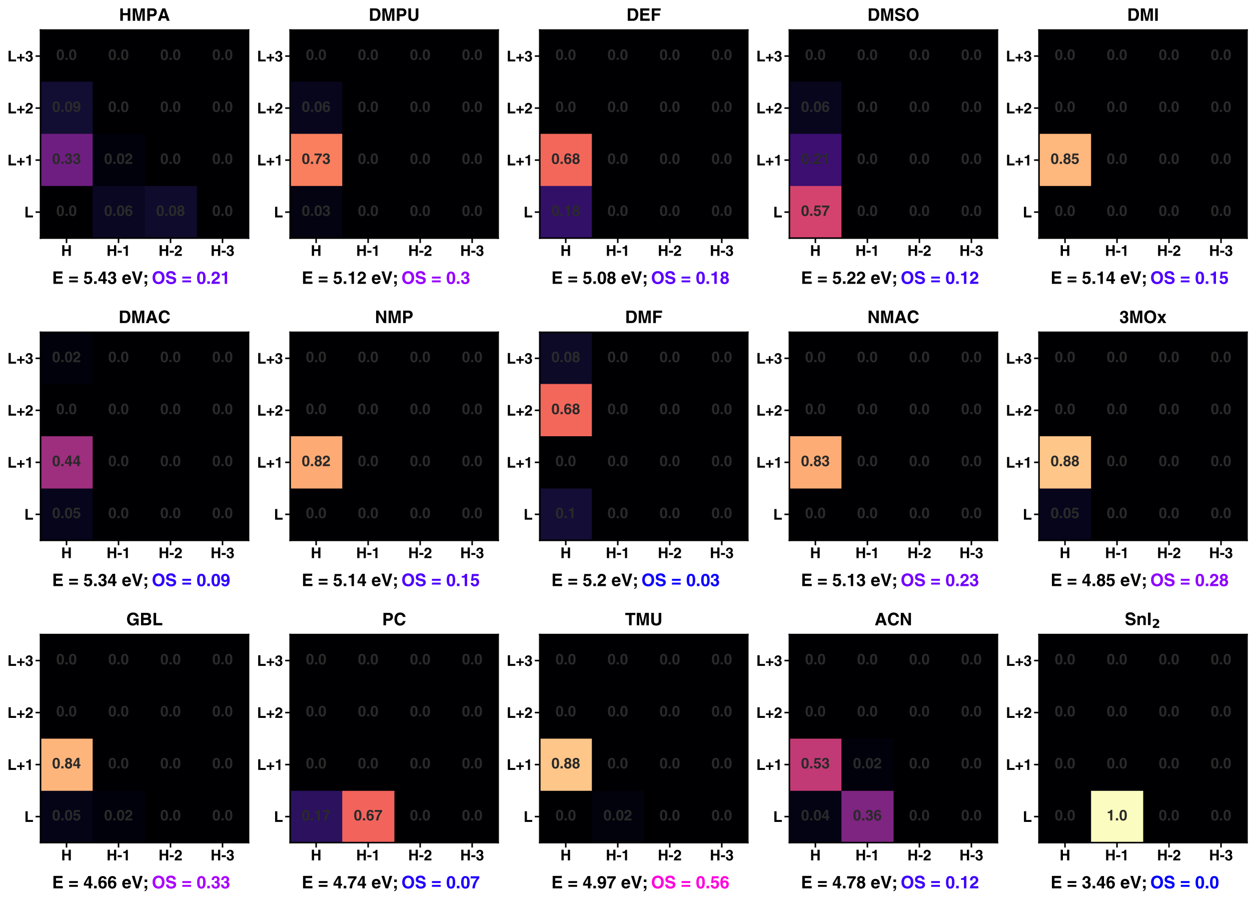

Figure S8: Composition of the second optical excitation of the 14 SnI_2M_4 complexes and the SnI_2 molecule in an implicit DMSO solution.The contribution $\in [0, 1]$ of the transition from the occupied (x-axis) to the unoccupied state (y-axis) is displayed in the corresponding grid square. H stands for HOMO and L for LUMO. For each transition, the energy E and oscillator strength (OS) are also shown.

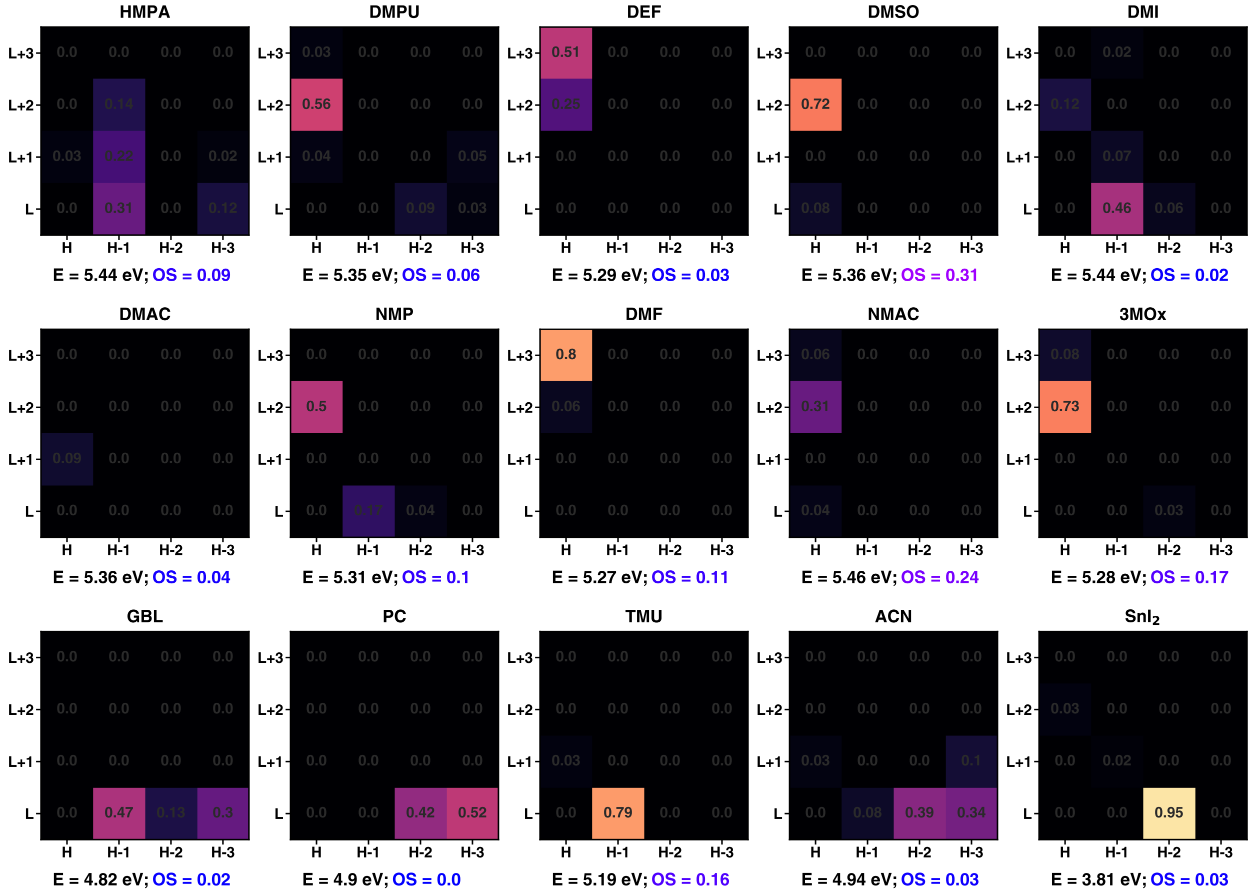

Figure S9: Composition of the third optical excitation of the 14 SnI_2M_4 complexes and the SnI_2 molecule in an implicit DMSO solution. The contribution $\in [0, 1]$ of the transition from the occupied (x-axis) to the unoccupied state (y-axis) is displayed in the corresponding grid square. H stands for HOMO and L for LUMO. For each transition, the energy E and oscillator strength (OS) are also shown.

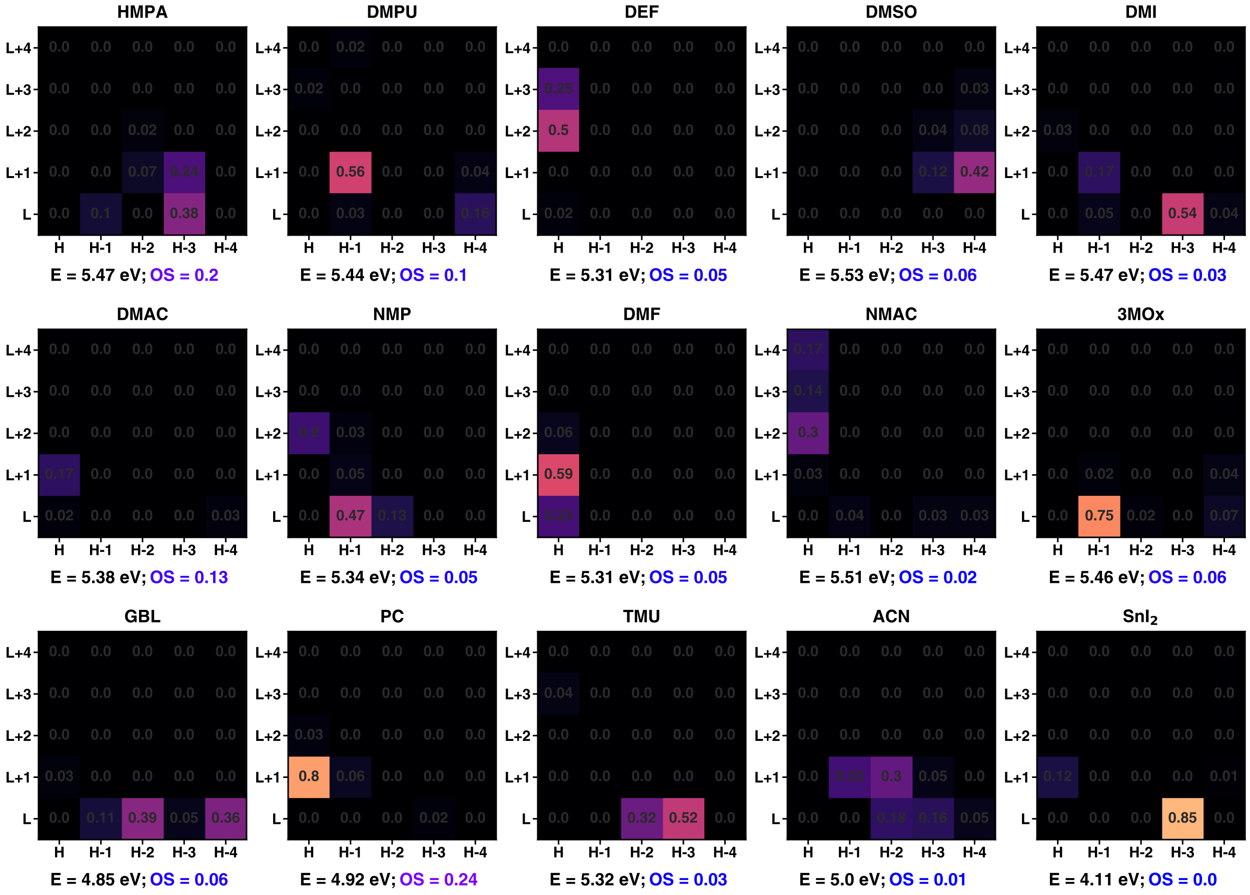

Figure S10: Composition of the fourth optical excitation of the 14 SnI_2M_4 complexes and the SnI_2 molecule in an implicit DMSO solution. The contribution $\in [0, 1]$ of the transition from the occupied (x-axis) to the unoccupied state (y-axis) is displayed in the corresponding grid square. H stands for HOMO and L for LUMO. For each transition, the energy E and oscillator strength (OS) are also shown.

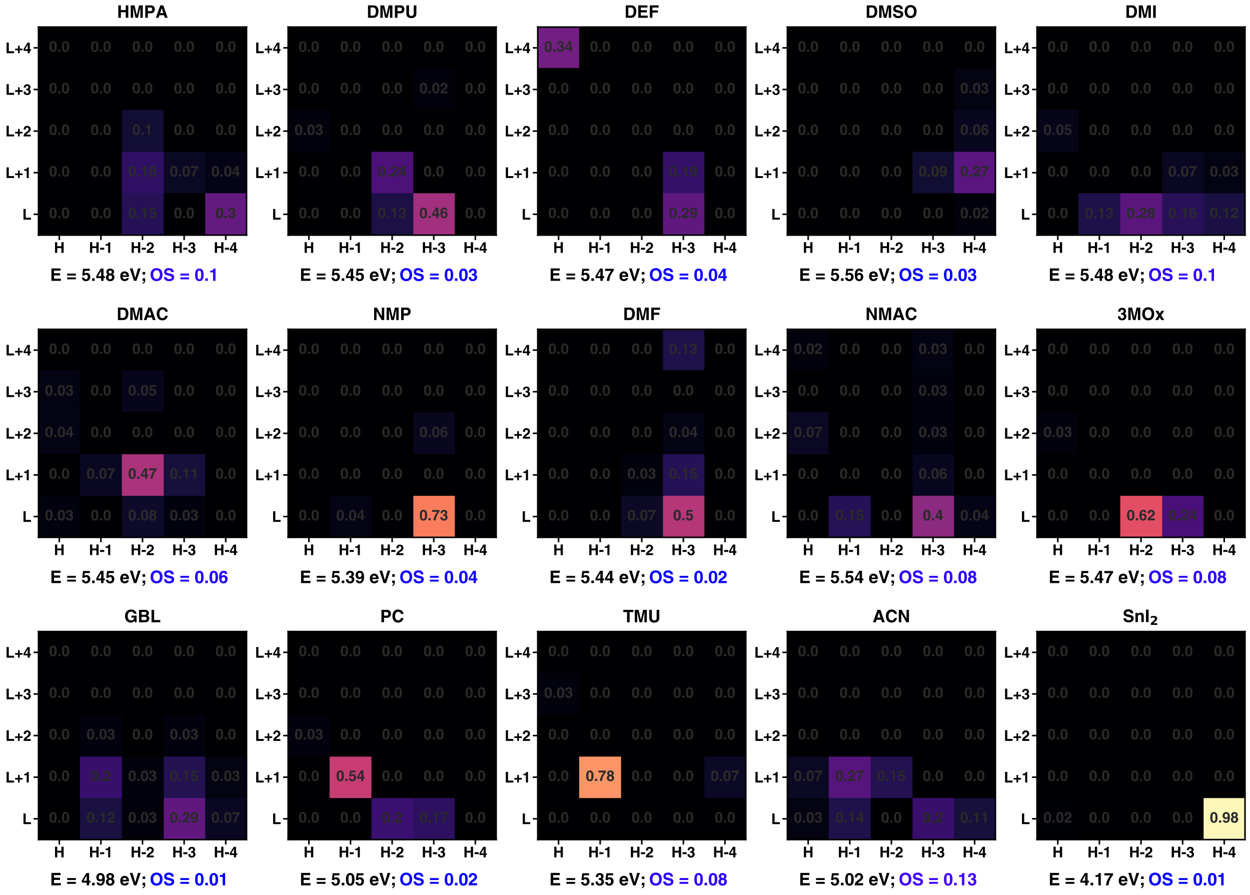

Figure S11: Composition of the fifth optical excitation of the 14 SnI_2M_4 complexes and the SnI_2 molecule in an implicit DMSO solution. The contribution $\in [0, 1]$ of the transition from the occupied (x-axis) to the unoccupied state (y-axis) is displayed in the corresponding grid square. H stands for HOMO and L for LUMO. For each transition, the energy E and oscillator strength (OS) are also shown.

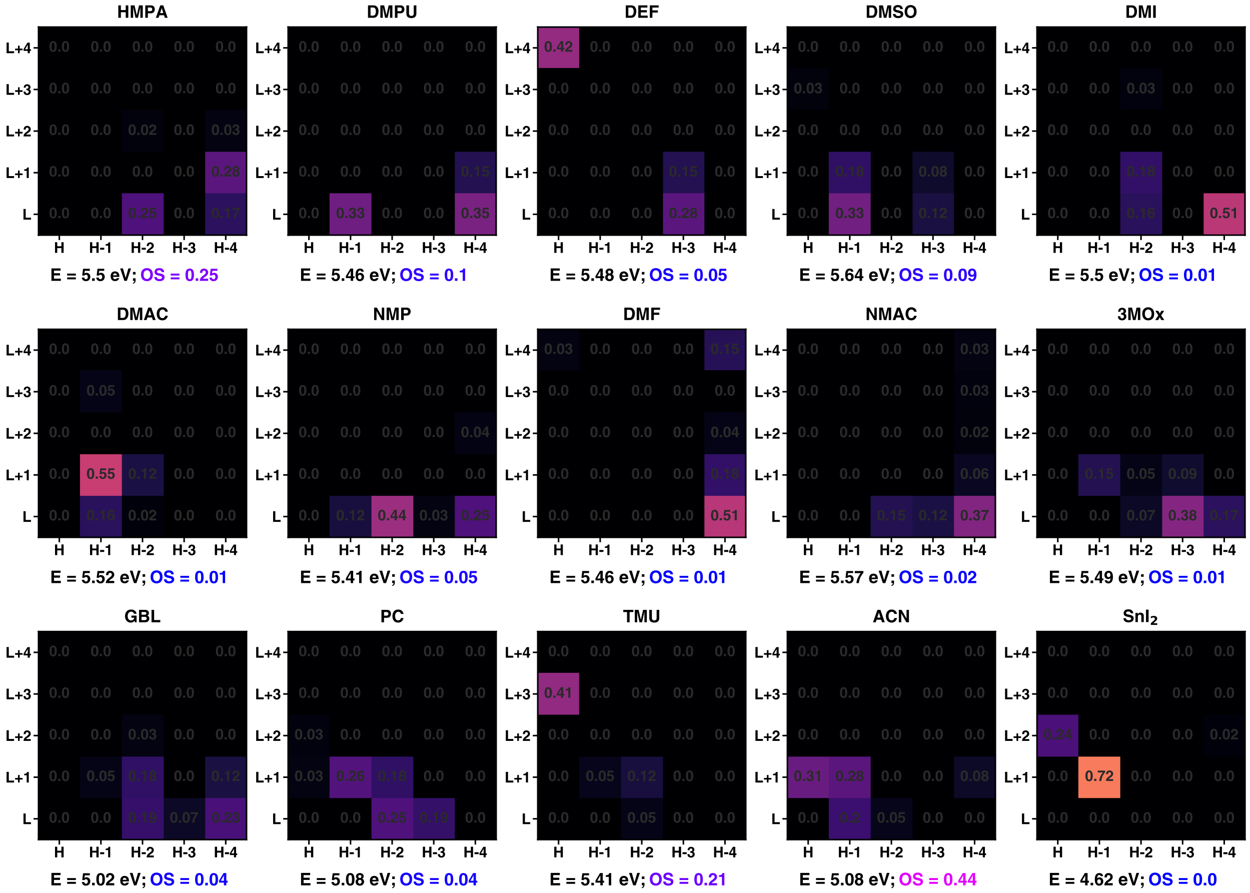

Figure S12: Composition of the sixth optical excitation of the 14 SnI_2M_4 complexes and the SnI_2 molecule in an implicit DMSO solution. The contribution $\in [0, 1]$ of the transition from the occupied (x-axis) to the unoccupied state (y-axis) is displayed in the corresponding grid square. H stands for HOMO and L for LUMO. For each transition, the energy E and oscillator strength (OS) are also shown.

References

(S1) Laurence, C.; Graton, J.; Gal, J.-F. An Overview of Lewis Basicity and Affinity Scales. *J. Chem. Educ.* **2011**, *88*, 1651–1657.

(S2) Miranda-Quintana, R. A.; Smiatek, J. Calculation of Donor Numbers: Computational Estimates for the Lewis Basicity of Solvents. *J. Mol. Liq.* **2021**, *322*, 114506.

(S3) Smiatek, J. Enthalpic Contributions to Solvent–Solute and Solvent–Ion Interactions: Electronic Perturbation as Key to the Understanding of Molecular Attraction. *J. Chem. Phys.* **2019**, *150*, 174112.

(S4) Ramirandaq, Ramirandaq/DonorNumberPrediction: DonorNumberPrediction. https://github.com/ramirandaq/DonorNumberPrediction (accessed 2023-01-02), **2020**.

(S5) Schier, R.; Conesa Rodriguez, A.; Valencia, A. M.; Cocchi, C. Formation of Lead Halide Perovskite Precursors in Solution: Insight from Electronic-Structure Theory. *Phys. Status Solidi B* **2021**, *258*, 2100359.
